# 胸骨骨髓细胞形态学在染色体核型正常获得性低增生性骨髓衰竭综合征且患者中的诊断价值

**DOI:** 10.3760/cma.j.issn.0253-2727.2022.11.008

**Published:** 2022-11

**Authors:** 佳 陈, 铁军 秦, 士强 曲, 丽娟 潘, 培红 张, 冰 李, 志坚 肖, 泽锋 徐

**Affiliations:** 1 中国医学科学院血液病医院（中国医学科学院血液学研究所），实验血液学国家重点实验室，国家血液系统疾病临床医学研究中心，细胞生态海河实验室，天津 300020 State Key Laboratory of Experimental Hematology, National Clinical Research Centre for Blood Diseases, Haihe Laboratory of Cell Ecosystem, Institute of Hematology and Blood Diseases Hospital, Chinese Academy of Medical Science & Peking Union Medical College, Tianjin 300020, China; 2 天津医学健康研究院，天津 301600 Tianjin Institutes of Health Science, Tianjin 301600, China

**Keywords:** 骨髓衰竭综合征, 低增生, 细胞形态学, 胸骨骨髓检查, Bone marrow failure syndrome, Hypocellularity, Cell morphology, Sternal bone marrow evaluation

## Abstract

**目的:**

探讨胸骨骨髓细胞形态学在基于髂骨穿刺骨髓细胞形态学和骨髓活检组织切片细胞形态学表现为染色体核型正常获得性低增生性骨髓衰竭综合征（BMFS）患者的诊断价值。

**方法:**

回顾性分析2014年6月至2019年1月中国医学科学院血液病医院（中国医学科学院血液学研究所）初诊的194例获得性低增生性BMFS患者的临床资料，结合胸骨骨髓细胞形态学结果重新诊断分型，分析各诊断分型患者的临床特征及预后差异。通过Logistic回归进行多因素分析，建立推荐该类患者进行胸骨骨髓细胞形态学分析的积分系统。

**结果:**

194例染色体核型正常的获得性低增生性BMFS患者，基于髂骨骨髓检查结果初步诊断为再生障碍性贫血（AA）152例，意义未明的特发性血细胞减少症（ICUS）29例，意义未明的克隆性血细胞减少症（CCUS）13例，结合胸骨骨髓细胞形态学结果后分别有42.8％（65/152）、24.1％（7/29）和30.8％（4/13）患者最终诊断为低增生型骨髓增生异常综合征（hypo-MDS）。胸骨骨髓检查结果最终诊断为AA与hypo-MDS患者总生存率差异有统计学意义（*P*＝0.005）。对结合胸骨骨髓检查结果最终诊断为AA和hypo-MDS两组患者临床特征、外周血血细胞参数和髂骨骨髓实验室特征进行单因素及多因素分析结果表明，年龄＞60岁（*OR*＝6.647，95％*CI* 1.954～22.611，*P*＝0.002，积2分）、NALP阳性指数≤160（*OR*＝2.654，95％*CI* 1.214～5.804，*P*＝0.014，积1分）、髂骨骨髓流式红系表型异常（*OR*＝6.200，95％*CI* 1.165～32.988，*P*＝0.032，积2分）及有DAT（DNMT3A、ASXL1、TET2）基因突变（*OR*＝4.809，95％*CI* 1.587～14.572，*P*＝0.005，积1分）均为影响染色体核型正常的获得性低增生性BMFS患者结合胸骨骨髓细胞形态学分析后诊断为hypo-MDS的独立预测因素。使用赤池信息准则（AIC）对模型进行预测效能评估，AIC＝186.1。当积分≥2分时诊断为hypo-MDS的特异性为91.7％，阳性预测值为80.6％。

**结论:**

染色体核型正常的获得性低增生性BMFS患者，仅通过髂骨骨髓细胞形态学和骨髓活检组织病理学可能产生误诊。我们提出一项积分系统，当积分≥2分时强烈建议该类患者进行胸骨骨髓细胞形态学检查有助于明确诊断分型。

## Abstract (en)

**Objective:**

Diagnostic value assessment of sternal bone marrow cell morphology in patients with acquired hypocellular bone marrow failure syndromes（BMFS）characterized by normal cytogenetics.

**Methods:**

A total of 194 eligible patients with an acquired hypocellular BMFS pre-sternum diagnosis in Institute of Hematology and Blood Diseases Hospital, Chinese Academy of Medical Science & Peking Union Medical College from June 2014 to January 2019 were reviewed. Sternal bone marrow evaluation was performed, and a post-sternum diagnosis was made. Clinical characteristics and overall survival（OS）were then compared among patients with different post-sternum diagnosis. Binary logistic regression was used to develop a predictive scoring system.

**Results:**

In 152 patients with pre-sternum AA diagnosis, 29 patients with a pre-sternum idiopathic cytopenia of undetermined significance（ICUS）diagnosis, and 13 patients with a pre-sternum clonal cytopenia of undetermined significance（CCUS）diagnosis, sternal bone marrow evaluation resulted in a change of diagnosis to hypocellular myelodysplastic syndrome（hypo-MDS）in 42.8％（65/152）, 24.1％（7/29）, and 30.8％（4/13）, respectively. Patients with a post-sternum hypo-MDS diagnosis showed a significant difference in OS compared with patients with a post-sternum AA diagnosis（*P*＝0.005）. Patients with ICUS/CCUS showed no difference in OS compared with AA and hypo-MDS（*P*＝0.095 and *P*＝0.480, respectively）. A 4-item predictive scoring system to identify hypocellular BMFS patients that need sternal bone marrow evaluation was developed, including age > 60 years old（*OR*＝6.647, 95％ *CI* 1.954–22.611, *P*＝0.002, 2 points）, neutrophil alkaline phosphatase score ≤ 160（*OR*＝2.654, 95％ *CI* 1.214–5.804, *P*＝0.014, 1 point）, abnormal erythroid markers evaluated by flow cytometry on iliac bone marrow（*OR*＝6.200, 95％ *CI* 1.165–32.988, *P*＝0.032, 2 points）, and DAT（DNMT3A, ASXL1, TET2）genes mutation（*OR*＝4.809, 95％*CI* 1.587–14.572, *P*＝0.005, 1 point）. The Akaike information criterin（AIC）was 186.1.

**Conclusion:**

Patients with a pre-sternum acquired hypocellular BMFS diagnosis characterized by normal cytogenetics may not reach accurate diagnostic categorization without sternal bone marrow cell morphology evaluation, which could be considered a diagnostic tool for this patient population. A predictive scoring system was developed, and when the total score is ≥ 2 points, sternal bone marrow evaluation should be performed for accurate diagnostic categorization that is critical to optimal patient care.

骨髓衰竭综合征（BMFS）是源于造血干/祖细胞损伤的一组临床异质性疾病，可分为遗传性及获得性两大类[1]–[2]。获得性BMFS（aBMFS）主要包括再生障碍性贫血（AA）、阵发性睡眠性血红蛋白尿症（PNH）及骨髓增生异常综合征（MDS）。低增生性MDS（hypo-MDS）与AA患者的临床表现及实验室特征存在重叠，仅通过髂骨骨髓细胞形态学及骨髓活检组织切片等相关检查尚不能进行准确诊断分型[3]–[4]。成人骨髓造血机能呈向心性分布，不同部位骨髓有核细胞增生程度不同，以胸骨增生最为活跃[5]。本研究中，我们对同时进行了髂骨和胸骨骨髓形态学检测的病例进行比较分析，探讨胸骨骨髓细胞形态学在髂骨骨髓形态学和骨髓活检切片组织病理学表现为染色体核型正常获得性hypo-MDS患者的诊断价值，并构建需进行胸骨骨髓穿刺患者的预测积分系统。

## 病例与方法

1. 病例资料：本项回顾性研究纳入2014年6月至2019年1月于中国医学科学院血液病医院（中国医学科学院血液学研究所）MDS诊疗中心初次就诊的194例获得性低增生性BMFS患者，中位年龄41（11～81）岁，男107例（55.2％），女87例（44.8％）。纳入标准：①外周血未见原始细胞；②参考《血细胞形态学分析中国专家共识（2013年版）》[6]和《骨髓增生异常综合征中国诊断与治疗指南（2019年版）》[7]的形态学判断标准及发育异常的定义以及其他有助于形态学判断的相关检查（包括巨核细胞CD41免疫组织化学染色等）进行形态学分析，髂骨骨髓检查结果不满足MDS最低诊断标准；③髂骨骨髓组织切片有核细胞增生程度经年龄校正后为增生减低[8]，网状纤维染色≤1级；④PNH克隆阴性；⑤骨髓造血细胞常规染色体核型分析和（或）荧光原位杂交（FISH）结果正常；⑥骨髓造血细胞采用二代测序（NGS）法进行基因突变检测；⑦除外先天性BMFS、骨髓增殖性肿瘤相关骨髓纤维化、大颗粒淋巴细胞白血病、淋巴瘤及非血液系统肿瘤等可能导致骨髓增生减低或骨髓衰竭的疾病；⑧首次髂骨骨髓检查后，7 d内同时进行了胸骨骨髓穿刺涂片细胞形态学分析。

2. 诊断标准及定义：AA的诊断及分型参考2015年英国血液学会推荐的诊断标准[9]。根据2016年版WHO造血及淋巴组织肿瘤诊断标准对MDS进行诊断及分型[10]。低增生性MDS定义为：符合MDS诊断标准且骨髓活检有核细胞增生程度经年龄校正后判断为增生减低[3],[8]。根据修订的国际预后积分系统（IPSS-R）对hypo-MDS进行预后分组[11]。意义未明的特发性血细胞减少症（ICUS）及意义未明的克隆性血细胞减少症（CCUS）的诊断标准参照文献[7],[12]：①外周血1系或多系血细胞减少（≥4个月）；②排除MDS和其他已知可导致血细胞减少的原因；③ICUS无髓系肿瘤相关体细胞基因突变，CCUS外周血/骨髓存在一个或多个与髓系肿瘤相关的体细胞基因突变且等位基因突变频率（VAF）≥2％。通过患者的临床特征、髂骨骨髓形态学及骨髓组织切片病理学对患者进行初步诊断，结合胸骨骨髓形态学对患者进行最终诊断分型。

3. 靶向测序检测基因突变：取患者诊断时的髂骨骨髓单个核细胞提取DNA样本，采用Ion Torrent半导体测序平台进行测序。平均基因覆盖率98.1％，平均测序深度1 314×。测序后原始数据利用CCDS、人类基因组数据库（HG19）、dsSNP（v138）等数据库进行生物信息学分析，筛选具有病理意义的突变位点。具体方法参照文献[13]。

4. 随访：随访截至2021年6月30日。随访资料主要来源于住院病历、门诊病历及电话随访记录。对随访期间死亡的病例，按照病例记录或与患者家属电话联系确认。总生存（OS）按确诊日期至死亡日期或末次随访时间计算。

5. 统计学处理：非正态分布的计量资料以“中位数（范围）”进行描述，采用Mann-Whitney *U*检验进行组间比较；分类资料以例数（构成比）进行描述，采用Fisher精确概率法进行组间比较。采用Kaplan-Meier法绘制生存曲线并通过Log-rank检验进行组间比较。采用Logistic回归进行多因素分析，根据具有独立预测价值因素的回归系数并参考文献[14]建议的方法构建预测积分系统。使用赤池信息准则（AIC）对模型进行预测效能评估。采用受试者工作特征曲线（ROC）计算连续变量cut-off值。双侧*P*＜0.05为差异有统计学意义。应用SPSS 26.0及R4.0.2进行统计学分析，采用R4.0.2绘制桑基图及生存曲线。

## 结果

1. 髂骨、胸骨骨髓细胞形态学诊断分型：结合患者临床特征及外周血检查结果，依据髂骨骨髓细胞形态学结果，194例患者中诊断AA 152例（78.4％），ICUS 29例（14.9％），CCUS 13例（6.7％）。进一步依据胸骨骨髓细胞形态学结果，髂骨初步诊断为AA的152例患者中76例（50.0％）仍诊断为AA，65例（42.8％）诊断为hypo-MDS［MDS伴单系血细胞发育异常（MDS-SLD）23例、MDS伴多系血细胞发育异常（MDS-MLD）37例、MDS伴原始细胞增多（MDS-EB）5例］，5例（3.3％）诊断为ICUS，6例（3.9％）诊断为CCUS；髂骨初步诊断为ICUS的29例患者中7例（24.1％）诊断为hypo-MDS（MDS-SLD 2例，MDS-MLD 4例，MDS-EB1 1例）；髂骨初步诊断为CCUS的13例患者中4例（30.8％）诊断为hypo-MDS（均为MDS-MLD）（图1）。

**图1:**
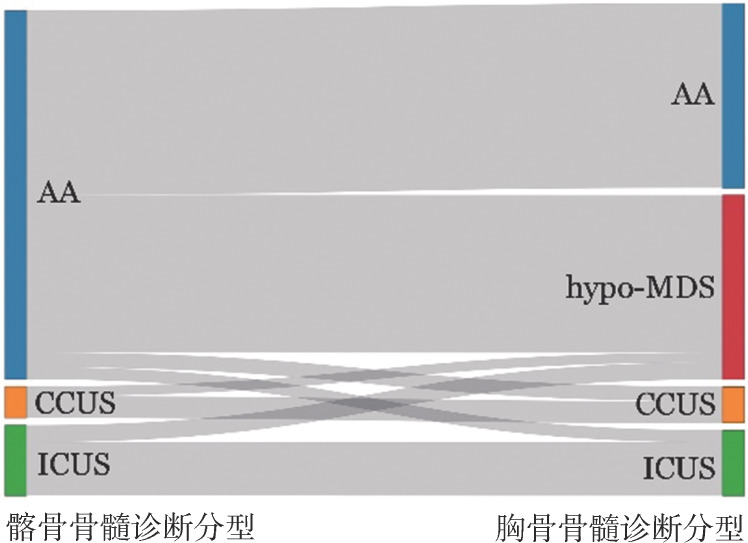
194例染色体核型正常的获得性低增生型骨髓衰竭综合征患者依据髂骨与胸骨骨髓结果的诊断分型变化 注 AA：再生障碍性贫血；CCUS：意义未明的克隆性血细胞减少症；ICUS：意义未明的特发性血细胞减少症；hypo-MDS：低增生性骨髓增生异常综合征

2. 临床与实验室特征比较：结合胸骨骨髓细胞形态学结果，诊断AA与hypo-MDS患者各76例；76例hypo-MDS患者均可进行IPSS-R预后评估，其中低危30例（39.5％）、中危44例（57.9％）、高危2例（2.6％）。两组患者临床特征、外周血血细胞计数、髂骨骨髓实验室特征及基因突变特征见表1。较AA组，hypo-MDS组年龄＞60岁患者比例（*P*＝0.010）、LDH水平（*P*＝0.031）、血清铁蛋白水平（*P*＝0.015）、髂骨髓系原始细胞表型异常比例（*P*＝0.030）、髂骨红系表型异常比例（*P*＝0.031）均更高，中性粒细胞碱性磷酸酶（NALP）阳性指数更低（*P*＝0.037）。AA组患者中，75例患者进行了基因突变检测，最常见的基因突变类型是EP300（8.0％）、DNMT3A（5.3％）、TET2（4.0％）及CREBBP（4.0％），hypo-MDS组，72例患者进行了基因突变检测，最常见的基因突变类型是EP300（9.7％）、TET2（9.7％）、ASXL1（6.9％）、DNMT3A（6.9％）、CREBBP（5.6％）及U2AF1（5.6％）。在DAT（DNMT3A、ASXL1、TET2）基因突变的患者中，AA和hypo-MDS组各有1例患者同时伴有DNMT3A和TET2突变。hypo-MDS组DAT基因突变比例显著高于AA组（22.2％对8.0％，*P*＝0.020）。

**表1:** 结合胸骨骨髓细胞形态学诊断为AA和hypo-MDS患者主要临床特征及实验室特征比较

临床及实验室特征	AA（76例）	hypo-MDS（76例）	统计量	*P*值
年龄[例（%）]			Fisher	0.010
＞60岁	5（6.6）	17（22.4）		
≤60岁	71（93.4）	59（77.6）		
IPSS-R分组				
低危	-	30（39.5）		-
中危	-	44（57.9）		-
高危	-	2（2.6）		-
LDH[U/L，*M*（范围）]	184（88~339）	197（112~380）	−2.163	0.031
血清铁蛋白[μg/L，*M*（范围）]	248（7~8 045）	543（9~3 766）	−2.441	0.015
髂骨骨髓表型异常[例（%）]			
髓系原始细胞表型异常	1（1.3）	7（9.2）	Fisher	0.063
红系表型异常	2（2.6）	10（13.2）	Fisher	0.031
NALP阳性指数[*M*（范围）]	177（86~330）	160（0~330）	−2.090	0.037
基因突变[例（%）] ^a^				
DAT突变	6（8.0）	16（22.2）	Fisher	0.020
DNMT3A突变	4（5.3）	5（6.9）	Fisher	0.742
ASXL1突变	0（0.0）	5（6.9）	Fisher	0.058
TET2突变	3（4.0）	7（9.7）	Fisher	0.203
U2AF1突变	1（1.3）	4（5.6）	Fisher	0.203
ZRSR2突变	0（0.0）	2（2.8）	Fisher	1.000
SRSF2突变	0（0.0）	1（1.4）	Fisher	1.000
EP300突变	6（8.0）	7（9.7）	Fisher	0.777
RUNX1突变	1（1.3）	3（4.2）	Fisher	0.360
CREBBP突变	3（4.0）	4（5.6）	Fisher	0.715
BCOR突变	0（0.0）	2（2.8）	Fisher	1.000
BCORL1突变	2（2.7）	1（1.4）	Fisher	1.000
DDX41突变	0（0.0）	1（1.4）	Fisher	1.000

注 AA：再生障碍性贫血；hypo-MDS：低增生型骨髓增生异常综合征；IPSS-R：修订版国际预后积分系统；NALP：中性粒细胞碱性磷酸酶。a：AA组患者中75例进行了基因突变检测，hypo-MDS组中72例进行了基因突变检测；-：不适用

3. 治疗与生存分析：结合胸骨骨髓细胞形态学，诊断为AA患者治疗方案为环孢素A联合促造血治疗［雄激素和（或）促红细胞生成素］；ICUS、CCUS及较低危hypo-MDS（IPSS-R≤3.5分）在AA治疗方案基础上加用沙利度胺；较高危hypo-MDS采用去甲基化药物（阿扎胞苷/地西他滨）治疗。中位随访55（2～84）个月，AA、ICUS/CCUS及hypo-MDS的3组患者5年OS率分别为（98.6±1.4）％、（92.5±3.5）％及（80.4±7.8）％。三组患者OS差异有统计学意义（*χ*^2^＝8.465，*P*＝0.015），hypo-MDS组OS率明显低于AA组（图2）。

**图2:**
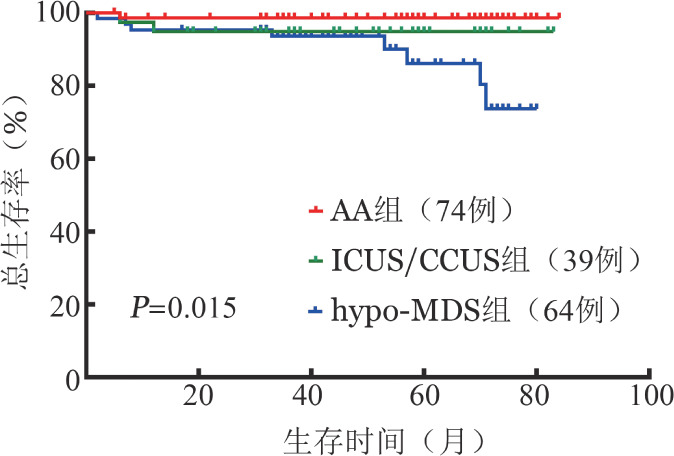
结合胸骨骨髓细胞形态学诊断为AA、ICUS/CCUS及hypo-MDS患者生存曲线比较 注 AA：再生障碍性贫血；ICUS：意义未明的特发性血细胞减少症；CCUS：意义未明的克隆性血细胞减少症；hypo-MDS：低增生型骨髓增生异常综合征

4. 建立推荐染色体核型正常的低增生aBMFS患者进行胸骨骨髓细胞形态学检查的预测积分系统：已有研究表明，ICUS/CCUS是可能发展为MDS等髓系肿瘤的前驱疾病之一，因此本研究仅基于最终诊断为AA和hypo-MDS患者构建预测模型。以胸骨骨髓细胞形态学诊断hypo-MDS为阳性事件，将表1中*P*＜0.05的因素纳入logistic回归模型进行多因素分析，结果显示年龄＞60岁（*OR*＝6.647，95％*CI* 1.954～22.611，*P*＝0.002）、NALP阳性指数≤160（*OR*＝2.654，95％*CI* 1.214～5.804，*P*＝0.014）、髂骨骨髓单个核细胞流式免疫表型分析红系表型异常（*OR*＝6.200，95％*CI* 1.165～32.988，*P*＝0.032）及有DAT基因突变（*OR*＝4.809，95％*CI* 1.587～14.572，*P*＝0.005）均为诊断hypo-MDS的独立预后因素。联合上述独立影响因素，依据各因素的回归系数赋分建立结合胸骨形态学诊断为hypo-MDS的预测积分系统，其中年龄＞60岁为2分，NALP阳性指数≤160为1分，髂骨骨髓单个核细胞流式免疫表型分析红系表型异常为2分，有DAT基因突变为1分，AIC＝186.1（表2）。ROC曲线计算总积分cut-off值为2分，当总积分≥2分时患者诊断为hypo-MDS的特异性为91.7％，阳性预测值为80.6％。

**表2:** 染色体核型正常的获得性低增生性骨髓衰竭综合征患者推荐进行胸骨骨髓细胞形态学检查的预测积分系统

因素	*β*	*OR*（95%*CI*）	*P*值	积分
DAT基因突变	1.570	4.809（1.587~14.572）	0.005	1
髂骨骨髓流式红系表型异常	1.824	6.200（1.165~32.988）	0.032	2
年龄>60岁	1.894	6.647（1.954~22.611）	0.002	2
NALP阳性指数≤160	0.976	2.654（1.214~5.804）	0.014	1

注：β：回归系数；DAT：DNMT3A、ASXL1、TET2；NALP：中性粒细胞碱性磷酸酶

## 讨论

aBMFS是后天获得性造血干/祖细胞质量异常的骨髓造血功能不良，导致外周血一系或多系血细胞减少的一组临床异质性疾病，主要包括AA、PNH和MDS[1]–[2]。10％～15％的MDS患者骨髓病理有核细胞增生程度经年龄校正后增生减低[4]，表现为hypo-MDS，与AA在临床及实验室特征存在重叠，而两者的临床转归又大相径庭。迄今为止，hypo-MDS与AA的鉴别诊断仍是血液科医师研究的重点和难点。2016年版WHO造血及淋巴组织肿瘤分类强调外周血或骨髓涂片原始细胞比例增多、髓系细胞发育异常比例≥10％及骨髓造血细胞检出具有MDS诊断价值的重现性染色体异常是诊断MDS的重要依据[10]。在染色体核型正常的aBMFS患者中，获得准确的骨髓原始细胞比例和髓系细胞发育异常比例对诊断显得尤为重要。虽然髂后上棘是目前骨髓穿刺和活检的首选部位，但胸骨骨髓穿刺仍是目前临床的检查方法之一。基于人体生理机能变化，成人骨髓组织随年龄增加逐渐被脂肪组织替代，造血机能呈向心性萎缩；而胸骨骨髓脂肪替代延迟，增生最为活跃[5]，可能通过胸骨骨髓穿刺获得更准确的细胞形态学信息。

1923年Seyfarth首先对胸骨骨髓穿刺技术进行了研究，1932年Forkner医生首次将该方法用于临床。二十世纪三四十年代，国内专家先后在中华医学杂志报道胸骨骨髓穿刺在黑热病的诊断和糙皮病所致贫血的临床应用[15]–[16]，还报道了北京协和医院26例中国健康成年男性胸骨骨髓形态学的研究结果[17]。随后胸骨骨髓穿刺逐渐在临床得以推广应用，国内小样本研究初步表明，与髂骨骨髓相比，胸骨骨髓形态学在AA与MDS的诊断分型及预后中有更高的准确性[18]。本研究的结果表明，髂骨初步诊断为AA的152例患者结合胸骨骨髓形态学检查后，50.0％的患者诊断得到了修正，其中42.8％的患者修正诊断为hypo-MDS；且修正诊断后的hypo-MDS患者与AA患者的OS有差异有统计学意义，进一步证实胸骨骨髓形态学在染色体核型正常的低增生性aBMFS患者的诊断价值。然而，胸骨骨髓穿刺的操作风险高于髂骨骨髓穿刺。英国血液学会的研究表明，骨髓穿刺的不良事件发生率为0.05％，其主要为胸骨穿刺所致主动脉破裂和心包填塞所致死亡[19]–[20]。与此同时，并非所有aBMFS患者均能通过胸骨骨髓穿刺获益。因此，有必要建立一项预测积分系统辅助临床医生识别那些更需要进行胸骨骨髓细胞形态学检查的患者，有助于提高诊断的准确率，同时减少不必要的胸骨骨髓穿刺。

我们的既往研究结果表明AA患者中位NALP阳性率及阳性指数均高于MDS患者，是鉴别AA和hypo-MDS的重要因素之一[21]。随着流式细胞术和二代测序技术在临床的应用，为AA和hypo-MDS的诊断和鉴别诊断提供了有力的证据补充。MDS流式细胞学国际白血病网络工作组（IMDSFlow）一项研究表明，流式检测红系细胞抗原CD36和CD71异常表达模式、联合CD71免疫荧光强度及CD117^+^红系组细胞比例可有效鉴别MDS和非恶性克隆性血细胞减少，其特异性达90％[22]。AA最常见的基因突变是PIGA（24.9％）、BCOR/BCORL1（19.6％）和DNMT3A（16.9％）[23]，而RNA剪切子基因突变及伴随DAT基因突变则是MDS最常见的基因突变模式，对MDS的诊断具有重要的临床意义（阳性预测值为0.86～1.0）[24]。我们通过对结合胸骨骨髓形态学最终诊断为AA和hypo-MDS的两组患者主要临床特征及髂骨骨髓实验室特征比较分析，建立了推荐染色体核型正常的低增生aBMFS患者进行胸骨骨髓细胞形态学检查的预测积分系统。该系统的预后参数包括诊断时年龄≥60岁、NALP阳性指数≤160、髂骨骨髓单个核细胞流式免疫表型分析红系抗原表达异常和伴随DAT基因突变。这些参数均可通过安全性更高的外周血或髂骨骨髓穿刺后检查获得，并通过上述积分系统选择出需要进行胸骨骨髓穿刺的患者，进而获得准确的骨髓原始细胞比例和细胞发育异常比例，既提高了诊断的准确性，又尽可能规避胸骨骨髓穿刺所带来的额外风险。

本研究的结果表明，对于染色体核型正常的低增生性aBMFS患者仅通过髂骨骨髓细胞形态学和髂骨骨髓活检组织病理学等检查容易误诊，当推荐胸骨骨髓形态学检查积分≥2分时强烈提示该类患者需进行胸骨骨髓细胞形态学检查，有助于患者的准确诊断分型和预后判断，以便采取正确的治疗策略提高疗效。本研究为单中心回顾性分析，纳入样本量较少，可能存在病例选择偏倚和预测因素遗漏，未来需多中心、大样本量的研究进一步验证。
